# Users’ Concerns About Endometriosis on Social Media: Sentiment Analysis and Topic Modeling Study

**DOI:** 10.2196/45381

**Published:** 2023-08-15

**Authors:** Rahul Goel, Vijayachitra Modhukur, Katrin Täär, Andres Salumets, Rajesh Sharma, Maire Peters

**Affiliations:** 1 Institute of Computer Science University of Tartu Tartu Estonia; 2 Department of Obstetrics and Gynecology Institute of Clinical Medicine University of Tartu Tartu Estonia; 3 Competence Centre on Health Technologies Tartu Estonia; 4 Women's Clinic Tartu University Hospital Tartu Estonia; 5 Division of Obstetrics and Gynecology, Department of Clinical Science Intervention and Technology (CLINTEC) Karolinska Institutet Stockholm Sweden; 6 Department of Gynecology and Reproductive Medicine Karolinska University Hospital Stockholm Sweden

**Keywords:** endometriosis, latent Dirichlet allocation, pain, Reddit, sentiment analysis, social media, surgery, topic modeling, user engagement

## Abstract

**Background:**

Endometriosis is a debilitating and difficult-to-diagnose gynecological disease. Owing to limited information and awareness, women often rely on social media platforms as a support system to engage in discussions regarding their disease-related concerns.

**Objective:**

This study aimed to apply computational techniques to social media posts to identify discussion topics about endometriosis and to identify themes that require more attention from health care professionals and researchers. We also aimed to explore whether, amid the challenging nature of the disease, there are themes within the endometriosis community that gather posts with positive sentiments.

**Methods:**

We retrospectively extracted posts from the subreddits r/Endo and r/endometriosis from January 2011 to April 2022. We analyzed 45,693 Reddit posts using sentiment analysis and topic modeling–based methods in machine learning.

**Results:**

Since 2011, the number of posts and comments has increased steadily. The posts were categorized into 11 categories, and the highest number of posts were related to either asking for information (*Question*); sharing the experiences (*Rant/Vent*); or diagnosing and treating endometriosis, especially surgery (*Surgery related*). Sentiment analysis revealed that 92.09% (42,077/45,693) of posts were associated with negative sentiments, only 2.3% (1053/45,693) expressed positive feelings, and there were no categories with more positive than negative posts. Topic modeling revealed 27 major topics, and the most popular topics were *Surgery*, *Questions/Advice*, *Diagnosis*, and *Pain*. The *Survey/Research* topic, which brought together most research-related posts, was the last in terms of posts.

**Conclusions:**

Our study shows that posts on social media platforms can provide insights into the concerns of women with endometriosis symptoms. The analysis of the posts confirmed that women with endometriosis have to face negative emotions and pain daily. The large number of posts related to asking questions shows that women do not receive sufficient information from physicians and need community support to cope with the disease. Health care professionals should pay more attention to the symptoms and diagnosis of endometriosis, discuss these topics with patients to reduce their dissatisfaction with doctors, and contribute more to the overall well-being of women with endometriosis. Researchers should also become more involved in social media and share new science-based knowledge regarding endometriosis.

## Introduction

### Background

Endometriosis is a debilitating gynecological disease that affects many women throughout their life. It is most common (6%-10%) among women of reproductive age, but it has also been found in adolescents [1] and premenarcheal girls [2] with chronic pelvic pain. With a relatively low incidence of 2% to 5%, endometriosis is diagnosed in postmenopausal women receiving hormone replacement therapy, exposing these patients to a higher risk of malignant transformation [3]. Endometriosis is a well-known cause of infertility associated with many other conditions that impair the physical and mental health of women [4].

The pathogenesis of endometriosis is poorly understood, and options for the diagnosis and treatment of this disease are limited. Several recommendations have been made to researchers on which research directions to focus on, and it was also suggested that more consideration should be given to patients’ views on what topics endometriosis research and clinical priorities should focus on [5]. Survey-based studies have shown a clear difference between what scientists and patients think are the topics that require urgent attention. Treatment was the highest priority area for both women with endometriosis and clinicians and scientists, but although scientists found cause and pathology and diagnosis and screening to be the next most urgent topics, persons who experienced endometriosis found topics about education and awareness, emotional impact, and comorbid conditions to be more critical [6].

However, surveys have limitations because they are usually targeted, and the mere knowledge that one is participating in a research study, even if anonymously, may bias answers. In addition, to take measures to improve the quality of life of patients with endometriosis, knowledge of women’s daily life problems related to this condition would be needed. To share information and experiences with other women with the same symptoms and express their feelings and concerns, patients with endometriosis actively use social media platforms [7,8]. It has been shown that social media allows a subjective, qualitative, and complex view of endometriosis that is rarely considered clinically or even in research and that there is a predominant communication and information gap between doctors and patients [7].

The first studies were conducted recently to analyze the most frequent endometriosis-related discussion topics on social media platforms Facebook and Instagram. These studies revealed that social media use offers emotional and network support, information and education about the disease, and the possibility of sharing personal experiences and discussing medication and treatment [9-11]. These studies have also shown that a wider awareness of topics discussed on the internet among health care professionals can help to understand the needs and demands of patients with endometriosis. One social media platform that is rapidly gaining popularity is Reddit [12]. Most Reddit users are aged between 18 and 49 years [13], which overlaps with the age group most often diagnosed with endometriosis. In addition, there is no content length limit on Reddit, the users can follow and discuss posts based on their interests, Reddit can provide additional relevant information, and comments are more accurate compared with other social media platforms [14]. The first computational data mining of endometriosis based on the analysis of Reddit posts showed that the most prominent and semantically related terms in the community were *endo*, *pain*, and *doctor* [15]. Furthermore, concept analysis revealed that this community could play a pivotal role in decision-making for women looking for immediate solutions to their needs, particularly for those who experience daily complications or are uncertain about how to address their problems through other means [15].

### Objective

This study aimed to identify the dominant topics of Reddit posts on endometriosis to gain further insight into women’s concerns and unmet needs, which require more attention from researchers and clinicians.

## Methods

### Data Set Description

We gathered data from Reddit through the official Reddit application programming interface (API) [16]. API is a tool that facilitates the interaction between computer programs and web services. It enables real-time data collection by tracking the live stream of public posts. Python libraries such as The Python Reddit API Wrapper [17] also assist this task by providing functions to collect Reddit data. Reddit is organized in communities called subreddits, which share a common topic and a specific set of rules. Users subscribe to subreddits, which contribute to their news feed with new posts. Inside each subreddit, a user can post or comment on other posts and comments. Thus, the overall discussion under each post evolves as a tree structure that grows over time.

### Data Collection

We used Reddit’s official API and Python Reddit API Wrapper to collect posts, comments, and associated metadata from 2 endometriosis subreddits that host public content: r/Endo and r/endometriosis. Corresponding to each post, we collected information on the title of the post, body or textual content, unique post ID, time stamp when the post was made, author ID, upvote ratio, number of comments, and post type defined by the user while publishing the post. Since posts gather comments over a period following the time of sharing, we collected corresponding post comments. The data set used in this study spans from January 2011 to April 2022.

### Posts Preprocessing

We started our preprocessing by removing all URL’s as they did not include any useful information for text analysis. Subsequently, the posts were subjected to lowercasing. The emojis (eg, 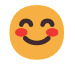), emoticons (eg, :D), numbers, mentions (@), and hashtags (#) were excluded from the posts because to their specific semantics in posts and overall text. In addition, all slang text was converted into its real meaning to understand the real context of the post. Furthermore, we proceeded with removing every punctuation mark and a variety of different stop words available in the *Natural Language Toolkit (NLTK)* package. We also performed the spell correction using *SpellChecker* package in Python (The Python Software Foundation). Using the *NLTK* package, we did the stemming and lemmatization of the post text. Lemmatization lowers the word forms to linguistically acceptable lemmas, whereas stemming reduces the word forms to stems to minimize size. As an example, the stem of the word “happy” is “happi,” but its lemma is “happy.” In particular, we used the *WordNetLemmatizer* function for lemmatization and the *PorterStemmer* for stemming of the *NLTK* package.

### Ethical Considerations

According to the policy of the Research Ethics Committee of the University of Tartu, ethics approval was not required for this study, as it involved the analysis of publicly available pseudonymous data. Consent was not obtained from individuals or Reddit, as the posts were available in the public domain and were made voluntarily from individuals who could not be identified.

### Sentiment Analysis

Sentiment analysis (SA) is a computational study of people’s opinions, attitudes, and emotions toward an entity [18]. An entity can represent individuals, events, or topics. The data set used in this study was collected directly from Reddit; therefore, the data set was not annotated. To overcome this issue, we followed an approach consisting of the following steps: (1) we manually annotated a randomly selected sample of 3000 posts; (2) then, we explored various models for SA (in particular, we used TextBlob [19], Vader [20], transformer-based model [21], and bidirectional encoder representations from transformers [BERT] model for sequence classification [22]); (3) using the trained models, we annotated the unannotated data; and (4) to measure the performance of the trained model, we selected 200 random samples from data annotated using trained models. We found that transfer learning with the BERT model for sequence classification performed best; therefore, we only discuss the details of this model.

First, we took an exploratory approach to analyze the posts. Initially, a pilot annotation was performed by 2 independent annotators, both annotating 100 observations to establish the relevant annotation rules. Posts were reviewed for 2 primary sentiments: positive (Does the post indicate an intention of being hopeful and confident and thinking about the good aspects of a situation?) and negative (Does the post indicate a sense of being unpleasant, depressing, or harmful and thinking about the bad aspects of a situation?). The third category was irrelevant posts, that is, posts unrelated to endometriosis or monosyllabic, with no discernible meaning. These 3 annotation categories were chosen to capture the possible opinion dimensions. After multiple discussions, both parties agreed to the finalized annotation rule set and guidelines.

Next, a set of 3000 randomly selected posts from the entire data set was annotated by 2 independent annotators. Annotators concluded 200 annotations with agreement, measured by Cohen κ of 0.70, which indicates substantial agreement. Following the annotation, both annotators worked together to adjudicate posts with differing annotations. The remaining 2800 posts were analyzed without comparing the annotators’ evaluations. Next, we used the annotated data for transfer learning.

Using the 3000 manually annotated posts, we fine-tuned the BERT model for sequence classification. BERT is a pretrained language model that helps machines learn excellent representations of text concerning the context. The 3000 manually annotated posts were split into an 80:20 training and test set. Specifically, 600 (20%) out of 3000 posts were used as the test set, and the rest were used as the training set. The evaluation metrics calculated for the test set were as follows: precision of 0.89, recall of 0.90 and *F*_1_-score of 0.89.

Subsequently, the fine-tuned BERT model was used to annotate the entire data set. To validate the efficiency of the annotated posts, 200 random posts were manually verified. The *F*_1_-score, precision, and recall values for the validation sample set were 0.91, 0.92 and 0.91, respectively, indicating that our model had been well trained, and we can rely on these labels to obtain further insights. The fine-tuned model is publicly available [23].

### Topic Extraction

Topic modeling is a statistical approach for grouping text documents based on the premise that each document is a function of latent variables called topics. Topic modeling methods are based on hidden variables (topics) that explain the similarities between observable variables (posts). The algorithm used for modeling topics was LDAMulticore [24].

The algorithm is a probabilistic generative model of topics, and the basic idea is that a post is composed of a random mixture of latent topics. Using the LDAMulticore model on the entire data set of the posts, we identified the optimal number of topics using a coherence score. Topic coherence measures scoring a single topic by measuring the degree of semantic similarity between high-scoring words in a topic. These measurements help distinguish between the topics. On the basis of the coherence score, the number of optimal topics in our data set was 48. Therefore, we extracted 48 topics from the data set, and LDAMulticore returned 10 words related to each identified topic (but not the topic’s title). The web-based graph to see the topics, keywords, and their respective distances is available on the link [25]. Furthermore, we assigned each post to a topic. As in the generative process, the algorithm assumes that a word belongs to a topic and that a document belongs to at least 1 topic. Under this premise, it is possible to correctly select the distribution of the topics per document.

## Results

### Data Overview

**Figure 1:**
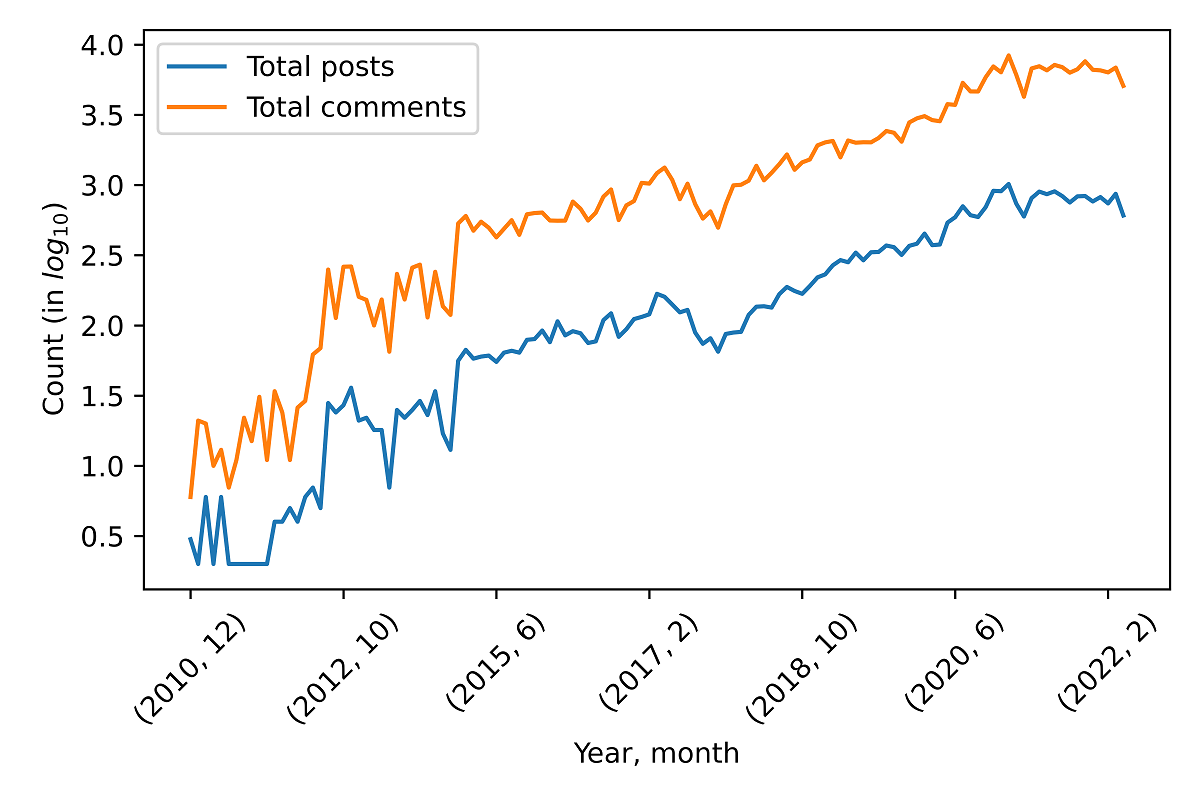
Data set timeline with the number of posts and comments. The x-axis represents the timeline, and the y-axis represents the number of posts in the log base 10.

Our data set contained 45,693 posts and 357,498 comments, with 18,099 unique users posting, 26,346 unique users commenting, and 31,144 unique users posting or commenting (Table 1). In total, 42.71% (13,301/31,144) of the users were found to write at least 1 post and comment. Figure 1 shows the timeline of the data set along with the number of posts and comments. The x-axis represents the timeline of the data set. For example, (2012, 10) represents October 2020. The y-axis represents the number of posts in the log base 10. The blue and orange lines represent the number of posts in log base 10 and the number of comments in log base 10, respectively.

**Table 1:** Data set statistics.

	r/Endo (period: January 2011 to April 2022), n	r/endometriosis (period: February 2014 to April 2022), n	Total (period: January 2011 to April 2022), n
Posts	29,322	16,371	45,693
Comments	228,709	128,779	357,498
Unique users (posts)	11,891	9089	18,099
Unique users (comments)	17,773	15,373	26,346
Unique users (posts and comments)	20,669	18,034	31,144
Unique users (wrote at least 1 post and comment)	8695	6428	13,301

For further investigation, we categorized Reddit posts into 11 categories using flair information in the post. These 11 categories are *Question*, *Rant/Vent*, *Surgery related*, *Tips and Recommendation*, *Medications and pain management*, *Good News/Positive update*, *Art, Memes and jokes*, *Infertility/Pregnancy related*, *Sex and intimacy related*, *Content warning/Graphic images*, and *Research*. The total number of posts and comments in each category is shown in Multimedia Appendix 1. The result revealed that posts belonging to categories such as *Question*, *Rant/Vent*, and *Surgery related* are higher in number. In contrast, we have fewer posts related to *Research*.

Next, we checked which category of posts had the highest number of comments. For proper comparison, we calculated the ratio of (the number of comments) divided by (the number of posts), as shown in Multimedia Appendix 2. We observed that categories such as *Art, Memes and Jokes*, *Content warning/Graphic images*, *Rant/Vent*, and *Sex and intimacy related* received more comments than average (*Overall*). Thus, we can infer that these categories produce high levels of user engagement. *Research* and *Good news/Positive News* attracted low user engagement.

### Endometriosis-Related Posts Express Mostly Negative Sentiments

Our SA revealed that most (42,077/45,693, 92.09%) of the posts were associated with negative sentiments, only 2.3% (1053/45,693) were annotated as positive, and 3.64% (1662/45,693) were irrelevant. Table 2 shows examples for each category. In addition, we report percentage sentiment, which is the net sentiment score (net sentiment=positive conversation − negative conversation) divided by the total number of posts in the data set. Therefore, the sentiment percentage for our data was −0.92.

Using the annotated posts, we checked whether any of the categories received more positive posts than negative ones. For proper comparison, we calculated the ratio of the number of positive posts divided by the number of negative posts, as shown in Multimedia Appendix 3. If this ratio is ≥1, the category has more positive posts than negative ones. In contrast, if the ratio is<1, there are more negative posts. We observed that all categories had a ratio of<1, indicating that there were primarily negative posts in the data set. The category *Good News/Positive update* had the highest positive-to-negative ratio, followed by *Art, Memes and jokes*, *Research*, and *Surgery related*, all having higher positive-to-negative ratios than *Overall* category (Multimedia Appendix 3); however, it must be emphasized that even the highest ratio (0.3 for *Good News/Positive update*) shows a very small proportion of positive posts among all posts.

**Table 2:** Examples of posts annotated as positive, negative, or irrelevant.

Annotation	Total posts (N=45,693), n (%)	Examples
Negative	42,077 (92.09)	“Post-op pain when urinating” “I believe my endo is returning after having total hysterectomy last year” “Pain. I am in pain.”
Positive	1053 (2.3)	“A breakthrough, non-invasive, blood test that aids in the diagnosis of active endometriosis!” “Second endo surgery was a success” “Finally got decent pain relief...”
Irrelevant	1662 (3.64)	“Date night!” “Exercising” “Alternative to peppermint tea for someone who hates both tea and peppermint?”

When manually annotating 3000 posts, we noticed that some posts were also made by individuals who were concerned about loved ones with endometriosis (15/3000, 0.5%). Some of these posts are most likely made by a man, for example, “A man with questions about his girlfriend with Endo,” and in some cases, the gender of the post author is not clearly identifiable, for example, “How to help a partner who suffers from endometriosis?”

### The Main Topics Related to Endometriosis

We used a topic modeling algorithm to identify hidden facets and more fine-grained topics of discussion under endometriosis. The number of optimal topics in our data set was 48. Columns 1 and 2 of Table 3 show the topic number and keywords returned by the LDAMulticore, respectively, and column 4 represents the number of posts that belong to each topic. We assigned appropriate topic names to each set of words that closely reflect the topic at an abstract level (column 3 of Table 3). While assigning the topic name, we observed that a few topics discussed the same thing, and we merged these topic keywords together. For example, topic numbers 10, 26, 41, and 43 returned by the LDAMulticore belong to *Laparoscopy/Surgery*. A total of 6 topics were also grouped under the common name *Questions/Advice*, all of which were primarily related to asking for information or advice on various topics related to endometriosis. We also noticed that although some topics such as *Doctor* and *Specialist* contain similar posts, they are rather different in tone, and we kept them as separate topics. For example, posts under the topic *Doctor* are mainly related to dissatisfaction with the doctors (“I think my doctor was wrong”), and posts under *Specialist* rather express satisfaction with getting a health care specialist appointment (“Endo Specialist Visit success!”).

After grouping the similar topics together, 27 independent topics remained. Sample post titles for each topic are provided in Table 4. We observed that *Laparoscopy/Surgery* is the most discussed facet, followed by *Diagnosis*, *Questions/Advice*, *Feelings*, and *Pain*. Simultaneously, it is evident that there may be some degree of overlap among topics. For instance, the post titled “Laparoscopy Thursday...Advice?” is assigned to the topic of *Questions/Advice*; however, it is also related to laparoscopy itself. Similarly, the post assigned to the *Laparoscopy*, titled “Shaving bikini line for lap?” seeks advice within its content before undergoing laparoscopy. The topic that brought together most research-related posts (*Survey/Research*) was the last topic in terms of posts. This topic encompasses various survey invitations, for example, “Seeking participants for survey research study,” sharing research outcomes (“Report and endometriosis patient survey results from BMI healthcare”), and expressing emotional dissatisfaction with the scarcity of endometriosis research (“F*** endo and f*** those not researching it”).

**Table 3:** Endometriosis-related topics on Reddit.

Topic	Keywords^a^	Topic name	Posts, n
10, 26, 41, and 43	lap, post, day, week, op, crazy, diagnostic, organ, accidentally, spontaneously, surgery, tomorrow, pregnancy, laparoscopic, scare, recently, ablation, fear, explain, pressure, laparoscopy, bleed, lose, endometrioma, end, anymore, heavy, concern, Orilissa^b^, burn, time, excision, surgery, recovery, hormonal, alone, terrify, live, supplement	Laparoscopy/​surgery	6696
4, 25, 32, 33, 34, and 45	help, manage, health, listen, surgical, scar, hell, rectal, resource, clear, advice, need, lupron^c^, people, understand, old, disease, put, condition, question, sex, flare, cause, Orilissa, life, medication, even, insurance, painful, else, weird, fatigue, ovulation, way, pain, pre, consider, bed, look, make, worried, eat, area, worry, shoot, taken seriously, impact, accept, think, see, use, bladder, woman, treat, loss, stress, bloated	Questions/advice	6409
0 and 36	bad, diagnose, tell, get, make, seriously, believe, trigger, unsure, man, diagnosis, schedule, nervous, fertility, love, drive, suggest, miscarriage, poop, underwear	Diagnosis	4906
5, 15, and 20	feel, birth control, stop, continuous, suddenly, likely, cover, even, totally, lol, scared, tired, blood, therapy, pelvic floor, return, morning, free, cancer, still, stage, depression, gas, date, die, pain, bring, colon, rd	Feelings/depression	2685
2, 6, and 38	pain, pelvic, give, do, med, research, sleep, wonder, intense, read, pain, right, management, side, expect, spot, constipation, emotional, large, hot, pain, relief, remove, medical, call, abdominal, study, let, switch, anxious	Pain	2544
18 and 22	get, cramp, sure, actually, check, successful, convince, already, thoracic, notice, symptom, say, wait, hurt, super, incision, adeno, worsen, potential, other	Symptoms	2424
14	period, long, normal, rant, recommendation, pain, leg, anxiety, cycle, feeling	Period	1794
11 and 39	try, possible, iud, ever, issue, low, body, worth, seek, chest, treatment, story, change, hormone, move, place, couple, risk, skin, guilty	Treatment	1417
8	experience, ask, stomach, similar, plan, big, partial, muscle, Depo-Provera^d^, discharge	Experiences	1003
21	doctor, painful, also, recommend, suffer, idea, home, information, clot, intercourse	Doctor	942
35	first, work, good, tip, ovary, chronic, removal, lot, soon, frustrate	Ovary	877
19	month, back, pill, come, well, due, pain, awful, liver, adenoma	Recurrency	828
24	go, sound, belly, never, friend, away, less, bit, confirm, kid	Confused	812
31	hysterectomy, want, share, little, hope, cope, maybe, newly diagnose, job, full	Hysterectomy	710
46	cyst, update, ovarian, miss, possibly, cry, grow, rupture, rib, deep	Cyst	662
3	take, bowel, support, Visanne^e^, show, adenomyosis, sign, relationship, second opinion, pant	Bowel	655
23	find, amp, option, yesterday, problem, side effect, uterus, point, early, sexual	Various problems	627
16	finally, answer, lady, suggestion, many, thank, infertility, dae, family, defeat	Positive titles	587
12	year, appointment, Mirena^f^, vent, last, flare up, follow, covid, infection, lie	Flare-up	490
27	deal, relate, test, ago, hip, positive, hour, pain, wish, swell	Coping	473
30	severe, next, exercise, tmi, frustrated, week, later, please, pee, gp	Exercise	442
42	specialist, surgeon, almost, different, appointment, happy, decide, hate, cure, laparoscopy	Specialist	413
28	ultrasound, result, talk, care, hard, visit, confused, progesterone, seem, flair	Ultrasound	387
17	nausea, struggle, diet, constant, control, horrible, able, medicine, head, weight gain	Nausea	365
13	bloat, bleeding, suck, child, prescribe, cbd, advocate, discuss, join, fellow	Bloating/bleeding	284
37	sick, baby, push, undiagnosed, regard, stand, implant, depressed, cervical, fall	Sick	168
1	survey, great, fuck, age, step, reason, currently, wits end, emergency, fine	Survey/research	136

^a^We excluded a few keywords from the topics because they were difficult to interpret. For example, in topic 10, we removed the keyword “cm,” which could refer to the size of an ovarian cyst in centimeters. Similarly, we removed “ve” from topic 45, which could be used as ″+ve ″ for positive or “−ve” for negative.

^b^A prescribed medicine to treat endometriosis-associated pain.

^c^A synthetic gonadotropin-releasing hormone.

^d^A hormonal medication.

^e^A hormone preparation for the treatment of endometriosis.

^f^A hormone-releasing intrauterine system.

**Table 4:** Topic manual labeling samples.

Topic name	Sample title
Laparoscopy/surgery	“Two weeks before first lap surgery”
Question/advice	“What does endometriosis pain during sex feel like?” “Advice for my first OBGYN^a^ visit?”
Diagnosis	“The specialist thinks I have endo, but isn’t scheduling to confirm”
Feelings/depression	“Feeling really sad and dejected” “Spiraling into depression”
Pain	“Pain relief suggestions that are not BC^b^ or NSAIDS^c^?”
Symptoms	“Endo symptoms while pregnant?”
Period	“Extremely uneven period flow”
Treatment	“Best progesterone treatment?”
Experiences	“Experiences with dietitians focused on endo?”
Doctor	“Frustrated with Doctors”
Ovary	“Surgery - removal of ovary”
Recurrency	“Can it come back?”
Confused	“It’s not endo, and now I’m not sure where to go.”
Hysterectomy	“Thoughts about a Hysterectomy”
Cyst	“Ovarian cysts - how fast has yours grown”
Bowel	“Do I have Endo of the bowel?”
Various problems	“Sexually active - source of your problem”
Positive titles	“A big thanks to this community”
Flare-up	“Unhealthy foods, flare ups?”
Coping	“How do you deal with skeptic bosses or coworkers?”
Exercise	“Does exercise help anyone with their pain?”
Specialist	“First appointment with the specialist tomorrow!”
Ultrasound	“Endometriosis showing on ultrasound”
Nausea	“Constipation, bleeding, nausea”
Bloating/bleeding	“Bloating on one side”
Sick	“Tired of Being Sick”
Survey/research	“International Survey - Endometriosis Research”

^a^OBGYN: obstetrician-gynecologist.

^b^BC: birth control.

^c^NSAIDS: nonsteroidal anti-inflammatory drugs.

A web-based graph for topic modeling is available in the study by Goel [25]. After downloading, any web browser may be used to interact with the topics and keywords. The graph can also be used to understand the overlapping of different topics through visualization.

## Discussion

### Principal Findings

In this study, we analyzed comments on the posts in r/Endo and r/Endometriosis on the Reddit social media forum. Since 2011, the number of posts and comments has steadily increased, indicating an increase in the awareness of endometriosis and the need for support from others with similar concerns. Endometriosis is a gynecological condition characterized by symptoms and complaints associated with the menstrual cycle, and women are reluctant to disclose their menstrual cycle disorders because it makes them susceptible to stigmatization [26]. Social media platforms allow women to gain information and share their personal experiences of endometriosis without identifying themselves. In addition, the overall search volume for endometriosis on websites is gradually increasing [27], and up to 76% of patients with endometriosis use social media for health information [8]. Moreover, endometriosis is the most popular minimally invasive gynecologic surgery topic on Instagram, with most authors being patients [28], and videos on TikTok primarily discussing personal experiences with endometriosis garner millions of views and likes [29].

Our SA showed that the vast majority of endometriosis-related posts were associated with negative sentiments, which is not surprising given the debilitating nature of the disease. There were no categories receiving more positive than negative posts, and the fact that the categories *Good News/Positive update* and *Art, Memes and jokes* received more positive posts compared with other categories is obvious because of their content; nevertheless, most of the posts in these categories are negative. The phenomenon that most health-related Reddit posts have a negative tone, expressing sadness, fear, and anger was described in a recent study by Maleki et al [30], which is believed to be related to users’ experiences in health care and the need for further information. Interestingly, *Research* and *Surgery related* posts have a higher positive-to-negative ratio than *Overall* categories, indicating that research-related information is sometimes shared with a positive mindset. The higher-than-overall proportion of positive posts in the *Surgery* category is likely because of cheering over referrals for laparoscopy (discussed in more detail in the next paragraph).

The number of posts in the category *Surgery related* was one of the largest, and the topic analysis revealed that the most discussed topic was *Laparoscopy/surgery* (6696/39,736, 16.85% of all posts), and the closely related topic *Diagnosis* (4906/39,736, 12.34% of posts) was in third place in the number of posts. Until recently, endometriosis diagnosis was confirmed only by laparoscopy [31], which is probably one of the reasons for the considerable time gap between the onset of symptoms and diagnosis [32]. Many posts reflected the patients’ high expectations for surgery, for example:

Nervous but also thankful about upcoming laparoscopy.

Finally approved for lap surgery for endo.

Women desire a diagnosis that explains their symptoms, providing reassurance that these symptoms are not caused by cancer or a figment of their imagination. They seek the opportunity to discuss their condition and receive better management strategies to control the symptoms. In addition, a diagnosis should help explain their absence from social and work engagements [33]. Finding noninvasive biomarkers is one of the research priorities [34], and the need for less invasive diagnostics was also mentioned in the posts, for example:

I feel like I won’t feel better until I get actually diagnosed and I just wish there was a less invasive option for diagnosis.

In contrast, the analysis revealed that there was a lack of sufficient professional information about the procedure, for example:

Can someone walk me through laparoscopy? I’m having my first one a week from Monday, and I’m scared.

Had excision last week. Stumbled across the subreddit, looking for postop advice. Now I’m terrified I’m not cured.

The second topic in the number of posts was *Questions/Advice* (6409/39,736, 16.12%). This topic covers a diverse range of themes where users are seeking to share their experiences and seek advice from others. Many questions are related to the experiences of other women with endometriosis, such as questions about sex life problems (“Sex Life with Endometriosis?”), symptoms (“People that had painful periods give your advice”), the causes of flare-ups (“Non-endo medication causing flare up?”). Information about good endometriosis specialists is also frequently sought. In many cases, the experiences of other women with similar problems can be useful, or at least mental help can be obtained from knowing that this issue is not only affecting them. The widespread use of social media as a platform for obtaining answers to endometriosis-related issues was also demonstrated in a survey-based study, which found that >80% of respondents had used social media to acquire information [7]. In contrast, there are also many topic initiatives in which it would be more appropriate to seek answers from health care professionals; for example, many posts ask for advice regarding an upcoming laparoscopy or postlaparoscopy recovery or recommendations regarding specific drugs. Asking these questions on social media can lead to quick answers; however, there is no control over the adequacy of the information. However, a study of educational posts on endometriosis Facebook pages showed that posts with educational content are mostly accurate, and common non–evidence-based claims were mostly related to alternative therapies [10]. There were also posts demanding professional advice (“Question for Healthcare Providers! [not personal medical advice]”); however, obtaining such advice can be complicated, as a study based on Instagram posts found that self-identified health care providers accounted for <3% of authors [28]. Similarly, the study by Blakemore et al [35] found that most Twitter and Instagram accounts on fertility-related social media were owned by patients, and these accounts were more influential than the physician, academic society, and fertility clinic accounts.

The other actively discussed topic was *Symptoms* (2424/39,736, 6.1%). Patients with endometriosis symptoms commonly report not getting adequate information from medical professionals [11] and absence of support from their doctors [36], a feeling also reflected in Reddit posts: “Symptoms—no help from doctors and feel crazy.” It has been shown that the worsening of endometriosis symptoms is the main factor for social media use, and among users, pain is one of the main symptoms [8]. We also noticed that 6.4% (2544/39736) of all posts belonged to the topic *Pain*. Women talk about the severity of the pain; how the pain interferes with their daily life (“I have to quit my job due to the pain”); ask for information about drugs (“Has anyone taken xxx for endo?”); and experiences with other methods, for example, alternative medicine and lifestyle for pain relief (“Have Functional/Natural Medicine practices helped you?”). *Pain* was also described as one of the dominant clusters in a previous endometriosis Reddit study, and this cluster was connected subsequently to the term *Symptoms* [15]. The mechanisms underlying endometriosis-associated pain are still unclear, and there are no effective treatment strategies for this symptom. Researchers have highlighted that pain is one of the prioritized topics that requires more attention [5].

Besides physical symptoms and complaints, women with endometriosis experience high levels of anxiety and depression [37]. In our study, *Feelings/Depression* was the fourth topic in terms of the number of posts, which also confirmed the relevance of mental health problems and the need for support in this community. Being able to freely express and share negative feelings on social media can have a therapeutic effect, as studies have shown that interacting with people who share similar negative emotional experiences can alleviate the effects of high stress [38]. In fact, the number of posts related to negative emotions is probably even higher, as many posts do not contain specific words indicating anxiety or depression but express apparent emotional stress in their content, for example:

I can’t carry on with this pain anymore.

Unfortunately, despite some positive responses, the category *Research* had the smallest number of posts and comments, demonstrating low user engagement. Researchers use social media to promote original research articles published in academic journals and disseminate the research output in various areas among the general public [39]. However, for example, there are very few posts by health care providers and physicians on Instagram [28]. In contrast, a study on Facebook pages showed that educational posts, particularly about the epidemiology and pathophysiology of endometriosis, are also popular, and most of the posts’ content was found to be evidence based [10]. Researchers should also make more use of social media opportunities to present research results to a wider audience and do so in a simple and understandable language to attract attention. However, posts must be accurate, and links to the publications should be included to give people the opportunity to learn more about the content and prevent misconceptions [40]. The topic analysis further stressed that the number of research-related posts was remarkably low, indicating both the low popularity of the topic among the endometriosis community and the low participation of researchers in these discussions. The greater need for endometriosis studies can also be guessed from the titles of some posts (“I don’t understand why endo isn’t talked about or researched more! And why don’t doctors know enough?!”). However, it should be noted that the actual number of posts related to research is likely somewhat higher. We observed several posts on this topic categorized under different topics, such as *Treatment* (“Research points to cannabis as an emerging treatment for endometriosis”) and *Questions/Advice* (“Biomedical Research-looking for help”).

### Comparison With Prior Work

Similar to previous endometriosis studies [10,11], our research indicated that women use social media to describe their struggles handling their daily problems, physical and emotional complications, and use of medical and alternative treatment and to get support from women with similar issues. Towne et al [10] found that *emotional support* comprised the largest number of posts on Facebook, and this category also had the largest overall engagement (69%). At the same time, educational posts received fewer likes and comments despite the relatively high number of posts, and among them, the subcategory “*Scientific article*” had only 2% engagement [10]. Similarly, *Research* attracted low user engagement in our study. However, a previous endometriosis social media study of Instagram and Facebook posts found that certain categories were covered less commonly than others; for example, only 2.8% and 0.2% of Facebook and Instagram posts contained disease-specific questions (*patient requests*), and the authors concluded that such intimate questions are more likely to be asked in closed groups and forums than publicly [9]. Our study, in contrast, showed that users on Reddit are actively looking for advice from their peers, with the largest number of posts and comments belonging to the category *Question* and 16.12% (6409/39,736) of posts under the topic *Question/Advice*. Our results also confirmed the findings of a recent study by Britt et al [15] showing that the Reddit community actively exchanges information about the etiology and symptoms of endometriosis and provides network support.

Endometriosis is a disease that affects almost exclusively women; however, a Facebook fan page developed by professionals to communicate reliable information on endometriosis and pelvic pain also comprised an audience of 11% male fans [41]. The number of posts probably made by male individuals was several times smaller in our study, a difference likely emerging from the available data of the given social media users. The study by Carneiro et al [41] collected page-specific metrics provided by Facebook, such as age, sex, and country of origin of their fans. Reddit enables the dissociative anonymity of users [42]; therefore, posts were annotated as potentially originating from men only if they directly indicated gender or contained certain keywords, that is, wife, girlfriend, and fiancée. It is important to note that, despite the chosen wording, it is not possible to definitively attribute these posts to men. It is widely known that endometriosis significantly affects the partners of women diagnosed with this condition. A recent study further highlighted the need for information and support resources specifically tailored to male partners [43].

### Limitations

Our study had some limitations. First, the largest number of Reddit users are from the United States, followed by Australia and India [13]. Health care systems vary from country to country; therefore, these results may not reflect the global situation. In addition, we analyzed only the posts in English. Second, as the pseudonymity of users on social media can lead to posts expressing negative emotions in particular, it may give a biased picture, especially regarding dissatisfaction with health care professionals. However, studies based on interviews have also shown similar trends [36].

### Conclusions

Our study confirmed that using social media to obtain and share information on topics to help manage endometriosis is a growing trend. A large number of posts with negative sentiment in the categories *Questions* and *Rant/Vent* indicate that women have both unanswered questions and a need to vent their frustrations about endometriosis. There is also a feeling of dissatisfaction with the role of health care professionals in dealing with the disease, suggesting that gynecologists and physicians should provide women with more reliable, necessary, and correct information about the aspects related to the diagnosis and treatment of this disease. However, our results also showed that although there is an overlap between research priorities and topics actively discussed in the endometriosis social media community, the content of posts is not related to scientific research but rather to the exchange of information within the community.

## Appendix

Multimedia Appendix 1 Reddit post categorization using flair information: the number of posts and comments are shown in each category. “Overall” represents the sum of all the posts.

Multimedia Appendix 2 Reddit post categorization using flair information: the ratio of comments and posts. “Overall” represents the ratio of comments and posts of all categories.

Multimedia Appendix 3 The ratio of positive and negative sentiments for Reddit post categories. “Overall” represents the ratio of positive and negative sentiment posts of all categories.

## Abbreviations

API: application programming interface
BERT: bidirectional encoder representations from transformers
NLTK: Natural Language Toolkit
SA: sentiment analysis

## Data Availability

The data set analyzed during this study was collected using the Reddit account application programming interface by our team. Owing to privacy concerns, the data are not publicly shared. However, the data set is available from the first author (RG) on reasonable request and can be accessible after signing the agreement for the use of data without violating privacy.
