# Supplementary figures and images for: Users’ Concerns About Endometriosis on Social Media: Sentiment Analysis and Topic Modeling Study
Source: J Med Internet Res. 2023 Aug 15;25:e45381. doi: 10.2196/45381 (PMC10466158; doi:10.2196/45381)

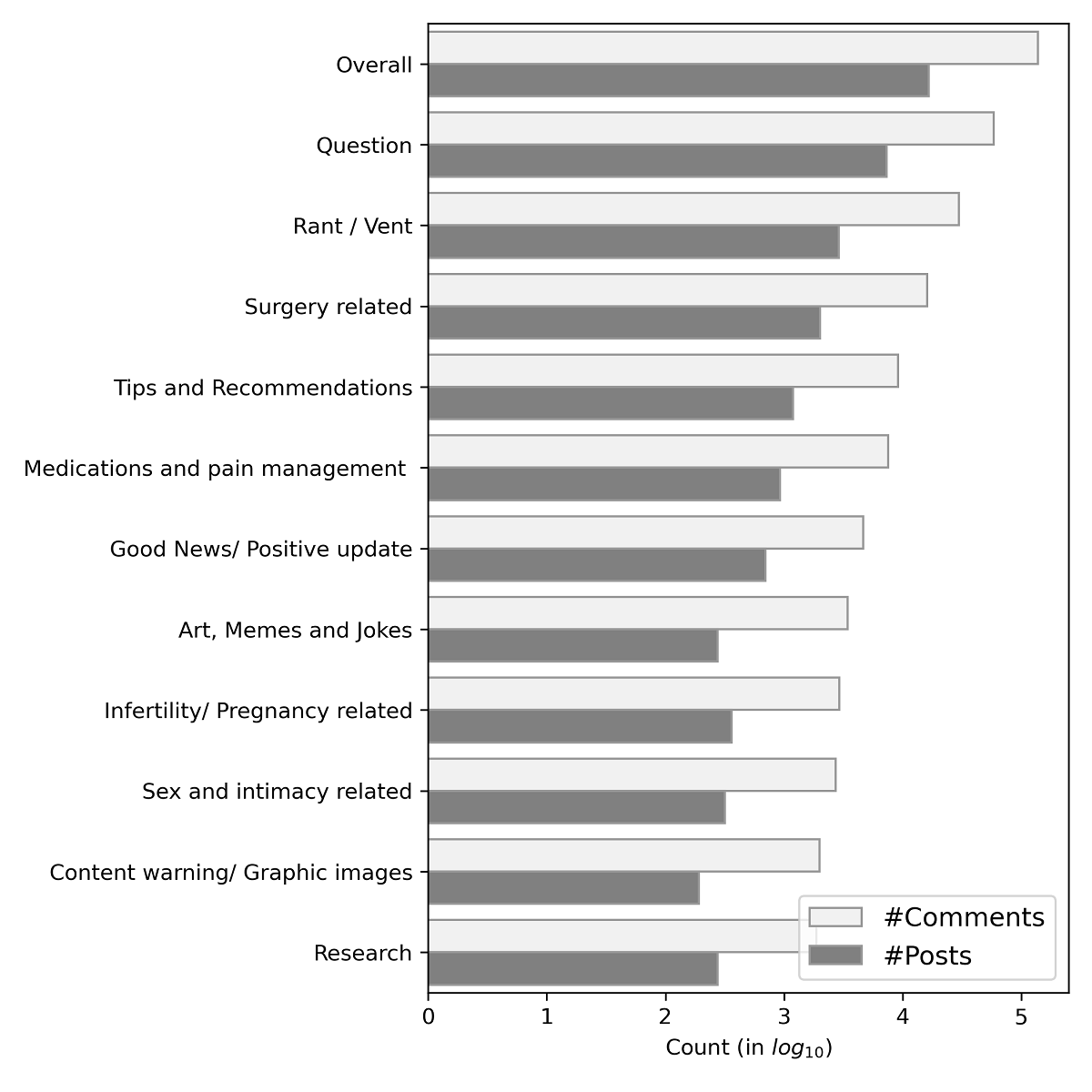

Supplement: Multimedia Appendix 1 [file jmir_v25i1e45381_app1.png]

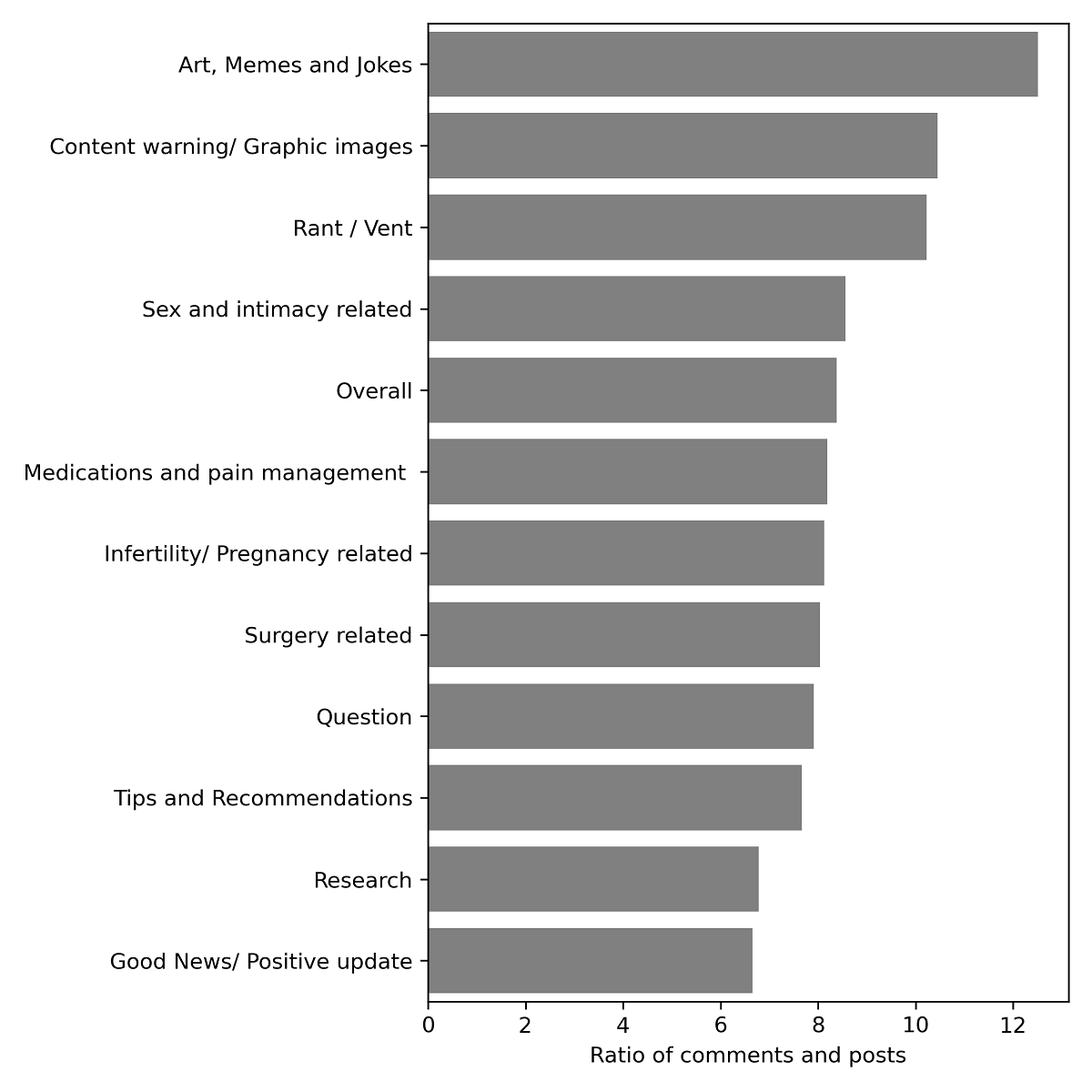

Supplement: Multimedia Appendix 2 [file jmir_v25i1e45381_app2.png]

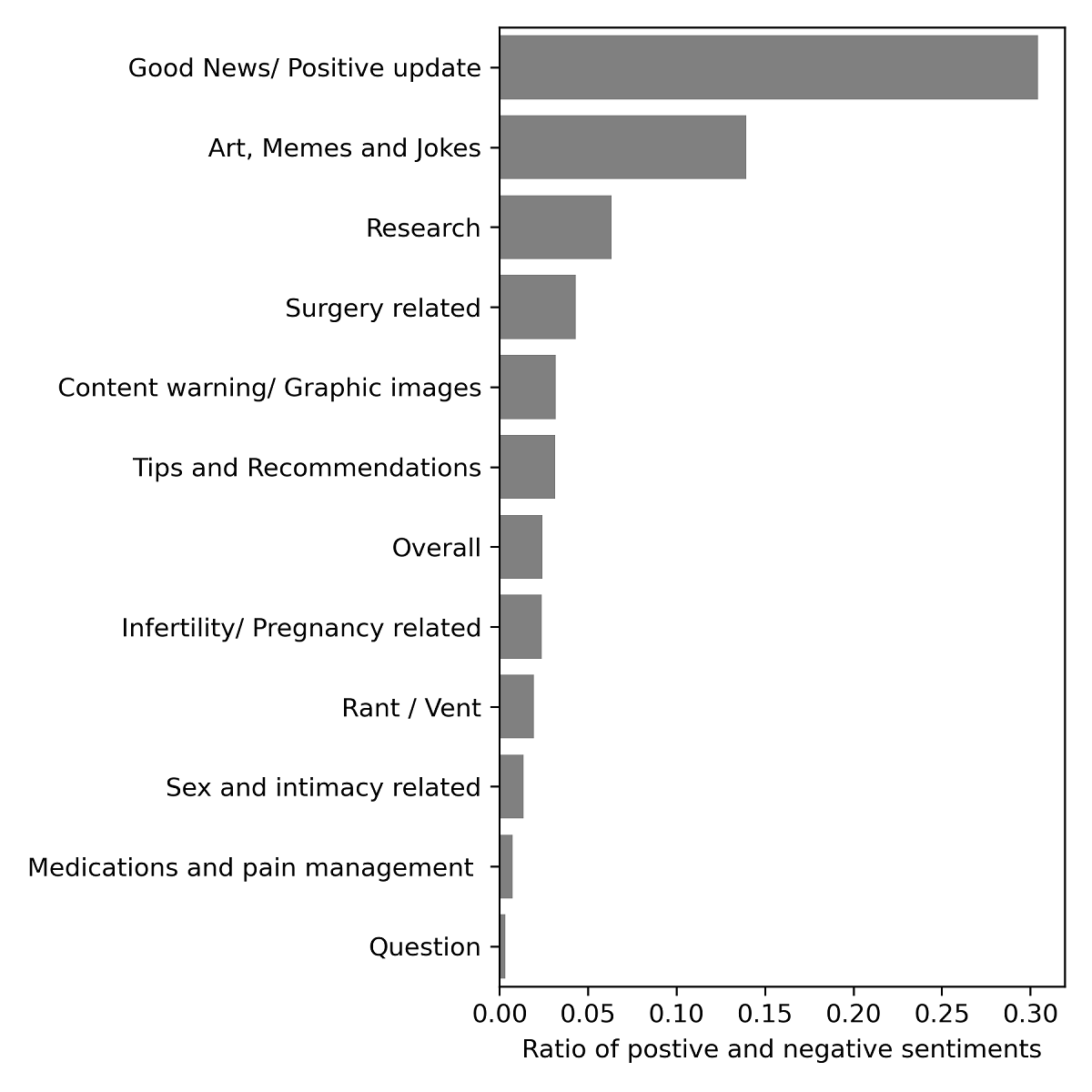

Supplement: Multimedia Appendix 3 [file jmir_v25i1e45381_app3.png]
